# DPCNet: A dual path cross perception network for small object detection in UAV imagery

**DOI:** 10.1371/journal.pone.0344091

**Published:** 2026-03-13

**Authors:** Linfeng Jia, Yafeng Zhu, Bin Li

**Affiliations:** School of Intelligent Manufacturing and Electrical Engineering, Guangzhou Institute of Science and Technology, Guangzhou, Guangdong Province, China; Instituto Politecnico Nacional, MEXICO

## Abstract

Small object detection in unmanned aerial vehicle imagery is challenged by tiny target scales, dense layouts, and cluttered backgrounds that blur fine details and destabilize multiscale representations. We present DPCNet, a single-stage detector that combines dual-path cross perception with deep and shallow feature interaction and a decoupled detection head. The Dual-Path Cross Perception block separates a detail stream and a semantic stream and performs gated bidirectional fusion, preserving edges while enriching context. The Deep and Shallow Feature Interaction block aligns features across levels through dynamic up-sampling and down-sampling and similarity-guided masking, which strengthens cross-scale consistency. The Dual-Path Decoupled Detection Head keeps classification and regression separate yet enables lightweight cross-branch channel and spatial guidance, and bounding-box regression adopts a geometry-sensitive Shape-IoU loss. Experiments on VisDrone2019 and HIT-UAV show consistent gains over the YOLO11n baseline: DPCNet improves mAP@0.5 by 2.0% and 5.1%, respectively, with higher precision and recall, especially for small, dense, low-light, and occluded targets. Despite modest computational overhead from cross-path interactions, the parameter count is reduced by about 45%, indicating a compact and robust solution for small object detection in challenging UAV scenarios.

## Introduction

Unmanned Aerial Vehicles (UAVs) have become essential tools in various critical applications, including security surveillance, agricultural pest management, traffic monitoring, environmental protection, and disaster rescue, due to their superior mobility, cost-effectiveness, and flexible deployment capabilities [1]. UAVs typically rely on visual sensors to collect environmental information by identifying and locating objects of interest, including people, vehicles, crops, or hazardous areas. This real-time perception capability enables UAVs to perform complex tasks autonomously. As a core component of UAV-based visual perception systems, object detection plays a crucial role in identifying and localizing multiple target categories in complex environments, providing the foundation for intelligent UAV applications [2]. Consequently, enhancing detection accuracy in UAV scenarios has emerged as a key challenge at the intersection of computer vision and UAV research [3,4].

Object detection in UAV imagery, however, faces two major challenges. First, because UAVs operate at high altitudes, objects on the ground appear small and highly variable in the captured images. This makes them susceptible to occlusion and background interference, leading to difficulties in maintaining stable representations with traditional feature extraction methods [5]. Furthermore, the small size of the objects results in the loss of critical edge information during feature down-sampling processes, which are common in many deep learning detection frameworks [6]. Second, varying background conditions, changes in lighting and weather, and UAV movement increase the uncertainty of detection tasks, which significantly impairs the robustness of existing methods across diverse environmental conditions [7,8]. These challenges are particularly exacerbated in extreme scenarios such as low-light conditions, dense object distributions, significant scale variations, and severe occlusions [9].

In response to these challenges, most existing research has focused on deep-learning-based object detection frameworks. Two-stage methods, such as Faster R-CNN, achieve outstanding detection accuracy through region proposal networks [10]. Single-stage models, such as You Only Look Once (YOLO) and Single Shot Multibox Detector (SSD), provide a balance between detection accuracy and speed through end-to-end learning. YOLO detects objects in a single pass through a convolutional network, whereas SSD combines multiple feature maps to detect objects at different scales [11,12]. Despite these advances, significant limitations persist in detecting small objects in UAV-based applications. For example, feature down-sampling operations in convolutional networks increase the receptive field but also lead to the loss of critical information for small targets [13,14]. Additionally, detection head design often struggles to balance classification accuracy and localization precision, especially for small objects with substantial scale variation [15]. Although Feature Pyramid Networks (FPN) and Path Aggregation Networks (PAN) improve multiscale feature fusion, they do not resolve the mismatch between deep and shallow features, which limits further gains in small object detection performance [16,17].

To overcome these limitations, we propose the Dual Path Cross Perception Network (DPCNet) for small object detection in UAV imagery. DPCNet targets weaknesses in feature extraction, fusion, and decoding with a set of specialized modules. Our primary contributions are as follows:

1. We introduce the Dual Path Cross Perception (DPCP) block, which decouples detail and semantic features and performs multi-stage bidirectional fusion. This design preserves fine-grained textures from shallow layers while integrating high-level semantics from deeper layers, enabling accurate representation of small objects even in cluttered or occluded environments.
2. To address cross-scale consistency, we propose the Deep and Shallow Feature Interaction (DSFI) block. It dynamically aligns hierarchical features using scale-aware modeling, thereby enhancing cooperation between deep and shallow features. This process improves the consistency and discriminability of cross-scale representations, which boosts small object detection in both dense and sparse scenes.
3. We also propose the Dual Path Decoupled Detection Head (DPD Head), which decouples classification and regression to reduce interference between them. By incorporating cross-channel attention, the DPD Head improves coordination between classification and localization, leading to more accurate localization, particularly in environments with significant scale variations and occlusions.

We evaluate DPCNet on the VisDrone2019 and HIT-UAV datasets. Experiments show that DPCNet improves mAP@0.5 by 2.0% on VisDrone2019 and by 5.1% on HIT-UAV relative to the baseline model, with higher precision and recall, especially on small and densely distributed objects. In addition, DPCNet demonstrates strong robustness and generalization in complex UAV scenarios, maintaining stable performance in challenging environments such as nighttime, dense scenes, and scenes with significant scale variations and occlusions.

## Related work

In recent years, research on small object detection in UAV imagery has mostly focused on optimizing architectures of YOLO-based detectors. To strengthen feature representation for small objects, work has primarily targeted the backbone, with design strategies including multiscale, lightweight, and dynamic fusion. For instance, the SMA-YOLO model significantly improves the retention of fine-grained features in aerial imagery by introducing a parameter-free Simple Slice Convolution module and multiscale fusion paths [18]. The HSFANet framework leverages a hierarchical scale-sensitive feature aggregation mechanism to align cross-layer features, thereby improving small object detection performance in complex scenes [19]. Additionally, the LGFF-YOLO network incorporates a Local–Global Feature Fusion Module, which notably improves small object detection accuracy while maintaining real-time performance [20]. The CBAM attention mechanism has been introduced into the C3 module of the backbone network to strengthen both channel and spatial feature representation [21]. The USF-DETR framework fuses spatial-frequency-domain feature modeling with multiscale geometric alignment, achieving semantic consistency of small-object features across feature maps of different scales and thus improving detection accuracy [22]. Taken together, these studies indicate that multiscale modeling and cross-layer semantic alignment in the backbone network are key strategies for improving detection performance in UAV scenarios. Meanwhile, recent semantic modeling studies in dense prediction also emphasize structured semantic organization and guided feature interaction, which offer transferable insights for detection by informing cross-level feature fusion and interaction design; for example, THE-Mask adopts a mask-classification paradigm for video semantic segmentation and enhances semantic utilization by introducing a hierarchical loss together with a temporal aggregation decoder for temporal modeling [23].

In detection head design, traditional YOLO detection heads perform classification and regression on shared features simultaneously, which often leads to task conflicts. To address this issue, the DMS-YOLOv5 model decouples the detection head and introduces a receptive field enhancement module, thereby effectively separating classification and localization features [24]. The dual IoU-aware decoupled head separates classification and regression tasks, which improves localization accuracy and convergence speed [25]. Additionally, the IA-YOLOv8 framework combines an intra-group multiscale fusion attention mechanism with an adaptive weighted feature fusion strategy, which significantly improves detection accuracy and robustness for small objects in complex aerial imagery backgrounds [26]. These studies indicate that decoupling the detection head and employing adaptive feature fusion are effective strategies for mitigating task conflicts and improving detection stability.

Feature fusion is another key aspect of optimizing small object detection performance. It helps bridge the representation gap between shallow high-resolution features and deep semantic features. AEFFNet enhances cross-layer feature consistency by introducing multi-axis frequency-domain and spatial attention modules [27]. CSFCANet employs a channel–space cross-attention mechanism to achieve feature complementarity and significantly improves the recognition of small-object boundaries [28]. EFA-YOLO improves detection efficiency and small-object recognition in UAV scenarios through lightweight feature aggregation and a dynamic spatial pyramid structure [29]. MDDFA-Net introduces a multiscale dynamic feature extraction module, which significantly boosts detection accuracy in complex infrared UAV scenarios [30]. MFFN improves small-object detection accuracy and robustness by integrating small-object feature layers and an enhanced TriC-IoU loss function [31]. The YOLO-SAR framework applies an improved YOLO architecture to UAV search and rescue tasks, demonstrating the feasibility of real-time small-object detection in resource-constrained systems [32]. Beyond UAV detection, cross-modal semantic modeling further suggests that mining semantic consistency can benefit robust fusion. For example, XMSNet explicitly mines cross-modal semantics by disentangling modality-shared and modality-specific cues and adopts attentive fusion with coarse-to-fine decoding [33]. In RGB-D salient object detection, CPNet designs cross-modal attention fusion and a progressive decoding strategy to gradually integrate low-level details under high-level semantic guidance [34].

Overall, these methods have advanced semantic consistency and cross-layer dynamic modeling, especially in areas such as multiscale feature fusion, attention mechanisms, and cross-layer feature alignment. However, most remain static or unidirectional enhancement strategies, while recent semantic modeling works suggest that bidirectional and structured interactions, including hierarchical semantic organization, progressive decoding, and consistency-guided fusion, can learn more stable semantic representations under complex variations. These strategies often struggle in UAV settings with large scale changes, cluttered backgrounds, and high object density, which degrades robustness and real-time performance. This limitation is particularly evident in challenging scenes such as nighttime operation, dense crowds, and severe occlusions, where stable detection is difficult to maintain.

## Proposed method

**Fig 1:**
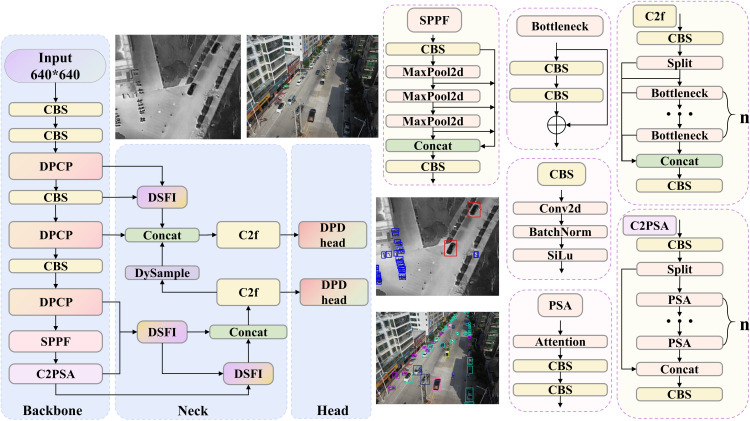
The overall structure of DPCNet.

To enhance the detection and localization accuracy of small objects in UAV imagery under complex conditions such as dense distribution, scale variation, occlusion, and low-illumination, this study proposes DPCNet, a single-stage detector trained end-to-end and built upon YOLO11n. YOLO11, released by Ultralytics, is an advanced end-to-end object detection framework that offers model sizes ranging from n to x (n/s/m/l/x). The architecture consists of a backbone, a neck, and a detection head. In the backbone and neck, YOLO11 employs the C3K2 module, which enhances feature map processing speed and richness through its dual-kernel and bottleneck design. Unlike previous versions, the backbone network incorporates the C2PSA module at the end, which improves feature attention by leveraging multi-head attention and feed-forward neural networks, optimizing both focus and gradient flow. The detection head utilizes depthwise separable convolutions, reducing computational costs while boosting operational efficiency. Therefore, the overall architecture of DPCNet also follows a hierarchical design composed of backbone, neck, and head networks to optimize feature extraction and multi-level feature fusion, as illustrated in Fig 1. We denote the backbone outputs as C3 to C8 and the corresponding pyramid features as P3 to P5. Here, Ck denotes the feature map from backbone stage k, and Pk denotes the fused feature map at pyramid level k.

In the backbone, the proposed DPCP block replaces the conventional C3K2 block. Through interaction between the detail and semantic paths, the DPCP block strengthens the representation of small objects, effectively preserving fine-grained details while improving multi-scale feature representation, especially in complex backgrounds. For the neck, DPCNet does not employ pyramid aggregation structures such as PAN. Instead, it introduces the DSFI block, which operates on three feature maps at different resolutions obtained after down-sampling. The DSFI block performs complementary enhancement between deep and shallow features at adjacent scales and integrates dynamic scale alignment and differential similarity masking, significantly improving the consistency and discriminability of hierarchical features. To further reduce computational overhead, the C2f module is used in the DSFI block. This module optimizes feature extraction by efficiently processing feature maps, thereby minimizing computational cost while maintaining high-quality feature representation. In the head network, DPCNet reduces the number of detection branches to two resolutions to better match small object characteristics. It also incorporates cross-feature interaction and adopts a DPD Head to decouple classification and regression. The DPD Head integrates channel cross gating and spatial attention to strengthen coordination between the two tasks. This design allows the classification branch to use boundary information from the regression branch, while the regression branch benefits from the semantic context of the classification branch, thereby improving overall detection performance.

## Dual path cross perception block

**Fig 2:**
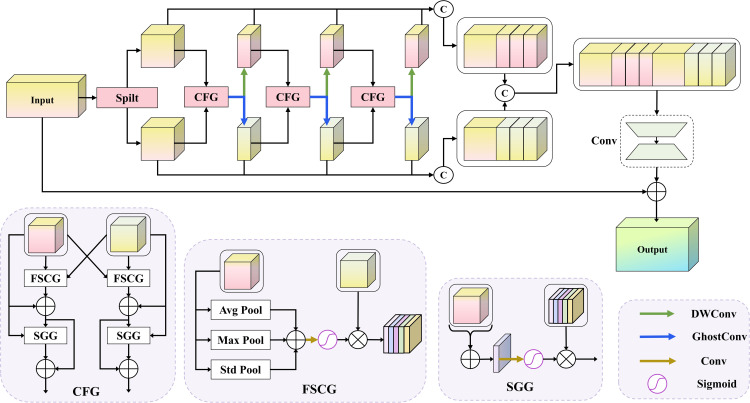
The structure of the DPCP block.

In UAV imagery, small objects often occupy only a few pixels, and their critical textures and edge details are easily lost during the down-sampling operations in convolutional networks, leading to feature degradation [35,36]. Moreover, complex backgrounds and scale variations require the network to capture local structural cues and maintain sufficient contextual awareness for semantic understanding across regions [37]. Although conventional convolutional networks can increase the receptive field by stacking layers, this approach often enhances global semantics at the cost of fine details and remains ineffective at modeling long-range dependencies [38]. To address these issues, we design the DPCP block to achieve dynamic feature enhancement through interaction between detail modeling and semantic modeling, as illustrated in Fig 2. The block uses two parallel feature extraction paths. The detail path stacks Ghost convolutions to retain high-frequency textures and edge information, whereas the semantic path applies dilated convolutions with increasing dilation rates to increase the receptive field and capture cross-object contextual information. Unlike one-time fusion strategies, the DPCP block performs multi-stage interactions between the two paths: semantic features guide structural enhancement, and detail features complement semantic understanding, yielding gradual bidirectional reinforcement between the streams.

The Cross Fusion Gate (CFG) governs the interaction. It comprises the Feature Statistics Channel Gate (FSCG) and the Spatial Guided Gate (SGG), which modulate features along the channel and spatial dimensions, respectively. In the FSCG, each path computes three global channel statistics (average, maximum, and standard deviation) and sums them to form a descriptor. We then pass the descriptor from one path through a lightweight one-dimensional convolution and a Sigmoid activation to produce channel gating weights for the other path, enabling residual cross-path modulation. As defined in Eq (1), the channel descriptors $P_{S}$ and $P_{D}$ are computed by aggregating global pooling statistics. As expressed in Eq (2) and Eq (3), the descriptors are passed through a convolutional layer and Sigmoid activation to generate gating weights, which are then applied in a residual manner to modulate the features of the other path. We define the FSCG-based cross-path residual gating as:


$$P_{S}\;=\;GAP(S)+GMP(S)+GSP(S),\;P_{D}\;=\;GAP(D)+GMP(D)+GSP(D).$$ (1)

$$g_{S}\;=\;\sigma \;\left(Conv1D(P_{D})\right),\;g_{D}\;=\;\sigma \;\left(Conv1D(P_{S})\right).$$ (2)

$$\hat{S}\;=\;S⊙\left(1+Reshape(g_{S})\right),\;\hat{D}\;=\;D⊙\left(1+Reshape(g_{D})\right).$$ (3)

where $S,D\in {\mathbb{R}}^{C\times H\times W}$ and $P_{S},P_{D}\in {\mathbb{R}}^{C}$ are channel descriptors. GAP(·) and GMP(·) denote global average and max pooling, respectively. GSP(·) denotes global standard-deviation pooling (computed over the spatial dimensions). Conv1D(·) is a lightweight 1D convolution operating on the channel descriptor, and σ(·) is the Sigmoid function. ⊙ denotes element-wise multiplication with broadcasting over spatial dimensions, and Reshape(g) reshapes $g\in {\mathbb{R}}^{C}$ to ${\mathbb{R}}^{C\times 1\times 1}$ for broadcasting. Note that $g_{S}$ is generated from $P_{D}$ and applied to modulate *S*, while $g_{D}$ is generated from $P_{S}$ and applied to modulate *D*.

This mechanism allows the semantic branch to guide the detail branch in emphasizing the structural cues, while the detail branch enhances the semantic branch by providing complementary local information. The residual gating mechanism preserves the stability of information flow and prevents over-suppression of the original features. The SGG further strengthens the complementarity between the two paths in the spatial dimension. Each path first sums the feature map along the channel axis to obtain a single-channel spatial map, then applies a lightweight convolution using a 5×5 kernel and a Sigmoid activation to produce a spatial attention mask. The mask is applied in a residual manner to highlight task-relevant regions and suppress background noise. This spatial gate is optional and can be activated to improve spatial discrimination or disabled to reduce computational overhead. After several stages of CFG-based interactions, the outputs of the two paths are concatenated along the channel dimension and unified using a 1 × 1 convolution. We introduce a residual connection between the input and the fused output to preserve information and stabilize training. By balancing feature preservation and representation enhancement, the DPCP block effectively strengthens small object perception and ensures consistent multi-scale feature representations, providing a strong foundation for subsequent feature fusion and detection.

## Deep and shallow feature interaction block

Current mainstream single-stage detectors, exemplified by the YOLO series, typically employ cross-level feature fusion structures built upon Path Aggregation Networks (PAN) to enable bidirectional information flow between high-level and low-level layers [39]. However, this fusion strategy depends heavily on repeated up-sampling and down-sampling operations. During down-sampling, deep features lose spatial details, while up-sampling diminishes the semantic richness of shallow features. Consequently, small object information degrades before fusion [40,41], and such loss cannot be effectively restored through multi-scale fusion in the feature pyramid structure [42]. Moreover, PAN often adopts static concatenation fusion schemes that fail to explicitly model the complementarity between deep and shallow features. This limitation prevents the network from maintaining semantic consistency and preserving details effectively across spatial and channel dimensions [43].

**Fig 3:**
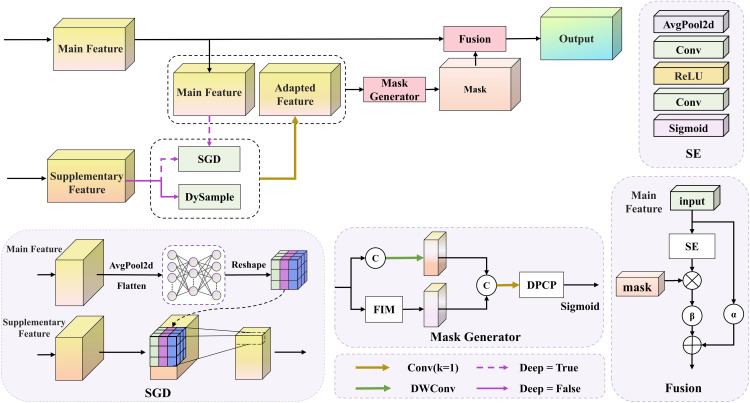
The structure of the DSFI block.

To mitigate passive feature degradation along the pyramid path, we introduce the DSFI block, as illustrated in Fig 3. DSFI directly extracts deep semantic features and shallow detail features from the backbone and establishes bidirectional interaction before fusion. Through dynamic alignment and explicit semantic-spatial collaboration, DSFI prevents information loss caused by conventional up-sampling and down-sampling. This design allows deep features to guide shallow semantic representations effectively, while shallow features provide complementary spatial cues to deep features.

Structurally, DSFI treats the enhanced feature as the main feature and the other as the supplementary feature, selecting alignment modes according to the interaction direction. When the shallow feature serves as the main branch, the deep feature is up-sampled using Dynamic Upsampling [44] to match the same resolution, ensuring semantic integrity. Conversely, when the deep feature acts as the main branch, the shallow feature undergoes Semantic-Guided Down-sampling (SGD). In this process, the deep feature is globally average-pooled and processed by a multi-layer perceptron (MLP) to generate dynamic convolutional kernels, which adaptively compress the shallow feature while retaining fine-grained, task-relevant information. After alignment, the main and supplementary branches are passed through a 1×1 convolution for channel matching to remove distribution bias and provide a unified representation for subsequent interaction.

As shown in Eq (4), the shallow and deep features are normalized using the $\ell_{2}$ norm at each spatial location (h,w).

Given the shallow feature $F_{s}\in {\mathbb{R}}^{C\times H\times W}$ and the deep feature $F_{d}\in {\mathbb{R}}^{C\times H\times W}$, both are normalized using the $\ell_{2}$ norm along the channel dimension at each spatial location (h,w):


$${\hat{F}}_{s}(c,h,w)=\frac{F_{s}(c,h,w)}{\sqrt{\sum_{c^{\prime }=1}^{C}F_{s}(c^{\prime },h,w{)}^{2}}+\varepsilon },{\hat{F}}_{d}(c,h,w)=\frac{F_{d}(c,h,w)}{\sqrt{\sum_{c^{\prime }=1}^{C}F_{d}(c^{\prime },h,w{)}^{2}}+\varepsilon }.$$ (4)

Here, the $\ell_{2}$ norms are computed over the channel dimension at each spatial location, and *ε* is a small constant introduced to prevent division by zero.

After normalization, pixel-wise cosine similarity is computed to measure the semantic consistency between the deep and shallow features at each spatial location (h,w), as defined in Eq (5):


$$sim(h,w)=\sum_{c=1}^{C}{\hat{F}}_{s}(c,h,w)\;{\hat{F}}_{d}(c,h,w).$$ (5)

DSFI removes weakly correlated regions and highlights discriminative areas through an adaptive threshold derived from global statistics. Let $\mu =\frac{1}{HW}\sum_{h=1}^{H}\sum_{w=1}^{W}sim(h,w)$ and $m=\max_{h,w}sim(h,w)$ denote the global mean and maximum over the spatial domain, and define τ=μ/(m+ε) based on sim(h,w).

When the similarity value at a given position exceeds *τ*, it is retained; otherwise, it is set to zero according to Eq (6):


$$\begin{array}{c} mask(h,w)=\left\{ \begin{array}{cc} sim(h,w), & \text{if }sim(h,w)>\tau ,\\ 0, & \text{otherwise}. \end{array}\right. \end{array}$$ (6)

After thresholding, DSFI broadcasts the spatial mask across channels as in Eq (7) to align with the original feature dimensions:


$$M_{sim}(c,h,w)=mask(h,w).$$ (7)

Here, $M_{sim}\in {\mathbb{R}}^{C\times H\times W}$ is obtained by replicating mask(h,w) along the channel dimension. It serves as a similarity mask that emphasizes semantically consistent regions while suppressing irrelevant background noise.

DSFI fuses this similarity mask with a difference mask along the channel dimension and aligns their dimensions through a feature alignment module. Next, DSFI applies the DPCP block to refine the fused mask. This refinement step promotes effective interaction between complementary and shared information across multi-scale representations. After refinement, DSFI activates the mask with a Sigmoid function to obtain the final interaction mask. During fusion, DSFI uses a channel attention mechanism to recalibrate the main branch features, emphasizing channels that contribute most to the detection task. It then multiplies the recalibrated features with the interaction mask element-wise, achieving cooperative enhancement of semantic and spatial information. A learnable coefficient *β* controls the strength of this enhancement. To keep the feature representation stable while maintaining fidelity, DSFI linearly merges the enhanced feature with the original input through a residual connection weighted by another learnable coefficient *α*. This mechanism keeps a dynamic balance between stronger expressiveness and structural integrity.

In the overall network, the C8 deep feature interacts with the C6 shallow feature at level P4, and the fused features are fed into the C2f block for small and medium object detection. Here, deep and shallow layers are defined based on the hierarchical relationship between the enhanced feature and its enhancement source. The feature being enhanced is regarded as the shallow layer, while the feature providing semantic guidance is regarded as the deep layer. The C3 feature, enhanced by information from C2 through the DSFI block, provides finer spatial detail and improved semantic consistency. Then, the up-sampled P4 feature fuses with the C4 shallow feature and the C3 feature enhanced by DSFI. Their concatenation is further processed by the C2f block to construct the P3 feature map, which combines rich spatial details and consistent semantics, enabling accurate detection of extremely small objects.

## Dual path decoupled detection head

In single-stage detectors, the classification task relies mainly on global semantic information to distinguish object categories, while the regression task focuses mainly on local boundaries and geometric structures to achieve accurate localization [45]. Traditional decoupled detection heads mitigate task conflicts by separating classification and regression, but full isolation disrupts the complementary relationship between semantics and structure. Consequently, the classification branch fails to leverage boundary priors from the regression branch to improve discriminative capability, and the regression branch fails to utilize semantic context from the classification branch to achieve stable localization [46]. Previous studies have shown that this information gap becomes especially apparent in small-object and occluded scenarios, often leading to category misclassification and boundary errors [47].

**Fig 4:**
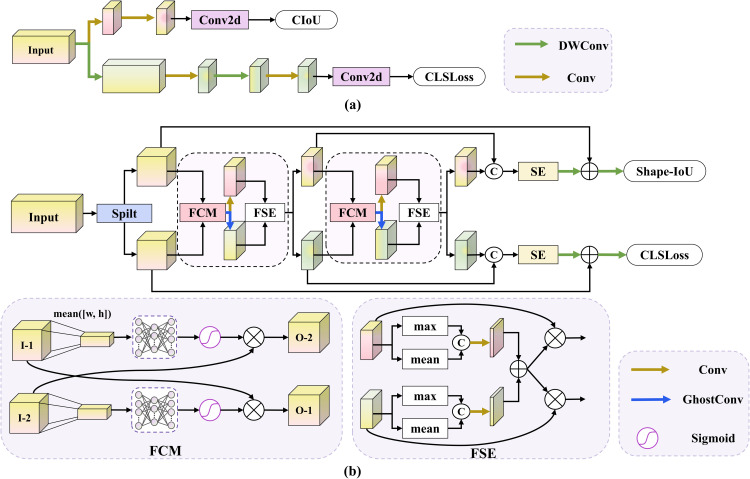
The architecture of detection head. **(a)** Baseline Head, **(b)** DPD Head.

To solve this issue, we introduce the DPD Head. As shown in Fig 4(a), the original detection head of the baseline model performs structural separation between classification and regression tasks. In contrast, Fig 4(b) illustrates that the proposed DPD Head explicitly incorporates a bidirectional interaction mechanism while maintaining task independence. Such a design decouples task-specific representations while explicitly bridging the semantic–geometric information gap through bidirectional interaction, which embodies the central idea of DPCNet.

In the overall structure, the input feature is divided along the channel dimension into a lightweight path and an expanded path, corresponding to the classification and regression branches, respectively. The classification path uses a Ghost convolution module to extract efficient semantic representations, while the regression path employs standard convolution to preserve geometric structure. This configuration maintains the representational independence of each task while ensuring efficient feature extraction. At each stage, the DPD Head introduces a Feature Channel Interaction Module to enable cross-path information exchange. Both paths are aligned by a 1×1 convolution before interaction. Let $F_{cls}$ and $F_{reg}$ denote the classification and regression features, respectively. The interaction module performs global average pooling on one path, passes it through a multilayer perceptron to generate channel weights, and writes the weights back to the other path to achieve cross-path guidance. The cross-path channel interaction is formulated in Eq (8).


$$\begin{array}{cc} F_{cls}^{\prime } & \;=F_{cls}+F_{cls}⊙Reshape\;\left(\sigma \;\left(MLP\;\left(GAP(F_{reg})\right)\right)\right),\\ F_{reg}^{\prime } & \;=F_{reg}+F_{reg}⊙Reshape\;\left(\sigma \;\left(MLP\;\left(GAP(F_{cls})\right)\right)\right). \end{array}$$ (8)

where $F_{cls},F_{reg}\in {\mathbb{R}}^{C\times H\times W}$ denote the aligned classification and regression features. GAP(·) denotes global average pooling over spatial dimensions, yielding a channel descriptor in ${\mathbb{R}}^{C}$. MLP(·) maps the descriptor to channel weights in ${\mathbb{R}}^{C}$ and σ(·) is the Sigmoid function. ⊙ denotes element-wise multiplication with broadcasting over spatial dimensions, and Reshape(·) reshapes a vector in ${\mathbb{R}}^{C}$ to ${\mathbb{R}}^{C\times 1\times 1}$ for broadcasting.The features $F_{cls}^{\prime }$ and $F_{reg}^{\prime }$ represent the updated classification and regression features after cross-path interaction.

After channel interaction, the DPD Head uses a Feature Spatial Enhancement (FSE) block to model spatial consistency. Each branch extracts both maximum and average spatial responses to generate spatial attention maps, followed by bidirectional interaction between the two branches. This process allows the classification branch to focus on potential target regions highlighted by the regression branch, while the regression branch refines its boundaries guided by the classification branch‘s semantics, thereby enhancing localization reliability. Channel interaction focuses on information composition, while spatial interaction focuses on information position. Together, they progressively integrate global semantics with local structure. Finally, each branch concatenates multi-stage outputs and performs intra-branch fusion through a 1×1 convolution, followed by channel attention to highlight task-relevant features. Residual connections are applied to enhance stability and feature fidelity. The refined features are then passed into the classification and regression heads, respectively, to generate the final class probabilities and bounding box parameters.

## Loss function

In the bounding box regression task, the Complete Intersection-over-Union (CIoU) loss improves optimization performance over traditional IoU, GIoU, and DIoU metrics by jointly considering the overlap area, center distance, and aspect ratio consistency between the predicted and ground-truth boxes. However, when the detected objects have small sizes, extreme aspect ratios, or very low overlaps, the IoU value of CIoU tends to approach zero, resulting in vanishing gradients and unstable optimization [48]. Moreover, its aspect ratio penalty term remains insufficiently sensitive when dealing with targets exhibiting large deformations or scale variations [49].

To enhance geometric sensitivity and shape adaptability in bounding box regression, the proposed DPCNet integrates Shape-IoU loss [50]. Shape-IoU improves CIoU by introducing two complementary components: a directionally weighted center distance term and a shape similarity penalty term. Together, they enable adaptive modeling of target geometry and scale variations. Following Shape-IoU, we summarize the formulation used in this work as follows.

The directionally weighted center distance term assigns anisotropic weights to the distance between the predicted box center $\left(x_{p},y_{p}\right)$ and the ground truth center $\left(x_{g},y_{g}\right)$, based on the width–height ratio of the ground truth box as defined in Eq (9):


$$w_{w}=\frac{2\;w_{g}^{\lambda }}{\;w_{g}^{\lambda }+h_{g}^{\lambda }\;},\;h_{h}=\frac{2\;h_{g}^{\lambda }}{\;w_{g}^{\lambda }+h_{g}^{\lambda }\;}.$$ (9)

Here, $w_{g}$ and $h_{g}$ denote the width and height of the ground truth box, and *λ* is a scale sensitivity factor that controls the strength of directional weighting. Let $w_{p}$ and $h_{p}$ denote the width and height of the predicted box. This design penalizes deviations along the shorter side more strongly and those along the longer side more weakly, improving geometric direction awareness. In particular, $h_{h}$ and $w_{w}$ are applied to the *x*- and *y*-directions, respectively, to emphasize deviations associated with the shorter side of the target box. The directionally weighted center distance is then formulated in Eq (10) as:


$$D_{c}=\frac{\;h_{h}\;(x_{p}-x_{g}{)}^{2}+w_{w}\;(y_{p}-y_{g}{)}^{2}\;}{\;c^{2}+\varepsilon \;}.$$ (10)

Here, *c* denotes the diagonal length of the smallest enclosing box that covers both the predicted and ground truth boxes, and thus $c^{2}$ is its squared value. The constant *ε* is introduced to avoid numerical instability when $c^{2}$ becomes small. Unlike the equal-weighted distance in CIoU, this formulation adaptively adjusts gradient distribution according to object shape, thereby improving optimization stability.

To capture differences in geometric proportions between the predicted and ground truth boxes, Shape-IoU introduces a shape penalty term. In what follows, $w_{p},h_{p}$ and $w_{g},h_{g}$ denote the predicted and ground-truth widths/heights, respectively. The discrepancy factors $\Omega_{w}$ and $\Omega_{h}$ are defined in Eq (11) as the normalized absolute differences between the predicted and ground-truth widths/heights. Here, max{·} provides scale-invariant normalization, and the directional weights $h_{h}$ and $w_{w}$ (defined previously) modulate the penalty according to the target aspect ratio. Based on these factors, the shape penalty $S_{c}$ is computed as in Eq (12).


$$\Omega_{w}=h_{h}\;\frac{\left|w_{p}-w_{g}\right|}{\max\{w_{p},w_{g}\}},\;\Omega_{h}=w_{w}\;\frac{\left|h_{p}-h_{g}\right|}{\max\{h_{p},h_{g}\}}.$$ (11)

$$S_{c}=(1-e^{-\Omega_{w}}{)}^{4}+(1-e^{-\Omega_{h}}{)}^{4}.$$ (12)

This term provides smooth gradients for minor discrepancies while rapidly increasing penalties for large mismatches. The fourth power further strengthens the constraint when shape differences become significant, ensuring stable convergence during training.

By combining the IoU overlap, directionally weighted distance, and shape penalty, the final form of Shape-IoU is expressed in Eq (13) as:


$$Shape\text{-}IoU=IoU-D_{c}-\frac{1}{2}\;S_{c}.$$ (13)

Here, IoU denotes the intersection over union between the predicted and ground truth boxes.

Shape-IoU offers more comprehensive geometric modeling and stronger directional sensitivity than CIoU, providing a robust constraint framework for bounding box optimization in complex scenes.

## Experiments and results

### Dataset and experimental environment

To comprehensively validate the performance of DPCNet across different modalities and complex UAV scenarios, we conducted experiments on two representative datasets, HIT-UAV [51] and VisDrone2019 [52]. These datasets cover diverse imaging conditions and scene densities, enabling a rigorous assessment of the model‘s small object detection capability under cross-modal settings.

The VisDrone2019 dataset, collected by the AISKYEYE team at Tianjin University, is a large-scale UAV-based visual dataset that includes both urban and rural scenes from fourteen cities. It contains ten object categories: pedestrian, person, car, van, bus, truck, motor, bicycle, awning tricycle, and tricycle, with more than 540,000 annotated instances. This dataset is characterized by small object sizes, dense distributions, severe occlusions, and class imbalance, and it is widely used to evaluate the performance of object detection algorithms in complex visible-light environments. In this study, the detection subset of VisDrone2019 was utilized, with 6471 images for training, 548 for validation, and 1610 for testing. The HIT-UAV dataset, released by Harbin Institute of Technology, is a high-altitude UAV infrared thermal imaging dataset designed to assess model robustness under low-illumination and noisy conditions. It consists of 2898 infrared images divided into training, validation, and test sets in a 7:1:2 ratio, corresponding to 2029, 290, and 579 images, respectively. The dataset covers representative UAV scenes such as campuses and parking lots and includes five annotated categories: Person, Car, Bicycle, Other Vehicle, and Don’t Care, comprising a total of 24,899 instances. Images were captured at altitudes ranging from 60 to 130 meters, featuring typical characteristics of small objects with weak texture cues.

All experiments were conducted on a server equipped with a single NVIDIA RTX 4090 GPU and a 20-core Intel Xeon Platinum 8470Q CPU, running Ubuntu 22.04. The implementation was based on PyTorch 2.5.1. All models were trained from scratch for 200 epochs with an input image size fixed at 640×640 pixels. The Stochastic Gradient Descent optimizer was employed with an initial learning rate of 0.01, dynamically adjusted using the Cosine Annealing strategy. The batch size was set to 16, and the weight decay coefficient was $5\times 10^{-4}$ to mitigate overfitting. To improve scale generalization, Mosaic data augmentation was applied during training.

### Evaluation metrics

To comprehensively assess the detection performance of DPCNet, we report Precision, Recall, Average Precision (AP), mean Average Precision (mAP), Parameters (Params), and Giga Floating Point Operations (GFLOPs). AP and mAP are computed following the COCO-style evaluation protocol [53] based on the Precision–Recall (P–R) curve under different IoU thresholds. The formulations are defined as follows in Eq (14):


$$Precision=\frac{TP}{TP+FP},\;Recall=\frac{TP}{TP+FN}.$$ (14)

Here, *TP*, *FP*, and *FN* denote True Positives, False Positives, and False Negatives, respectively. A detection is counted as a true positive if it matches an unmatched ground-truth box of the same category with an IoU above a given threshold; otherwise, it is a false positive, while unmatched ground-truth boxes are counted as false negatives. AP is the area under the P–R curve, expressed in Eq (15) as:


$$AP=\int_{0}^{1}P(R)\;dR.$$ (15)

In practice, AP is approximated by numerical integration over the discrete P–R curve obtained by ranking detections by confidence scores. By averaging AP over all *N* categories, mAP is obtained, as defined in Eq (16):


$$mAP=\frac{1}{N}\sum_{i=1}^{N}AP_{i}.$$ (16)

We report mAP@0.5 and mAP@0.5:0.95. mAP@0.5 uses a fixed IoU threshold of 0.5, whereas mAP@0.5:0.95 follows the COCO-style definition and averages AP over IoU thresholds from 0.5 to 0.95 with a step size of 0.05. To quantify model complexity, we also report Params and GFLOPs, which are computed for a single forward pass at an input resolution of 640×640.

In addition to accuracy and complexity indicators, we report deployment-oriented efficiency using runtime latency and throughput under single-image inference. Runtime latency is reported in milliseconds per image(ms/image), and throughput is reported as frames per second, denoted as FPS. To reflect practical UAV inference pipelines, we adopt an end-to-end timing protocol, denoted as E2E, which accounts for preprocessing, network inference, and post-processing. As shown in Eq (17), the total end-to-end latency is broken down into three components:


$$T_{total}=T_{pre}+T_{inf}+T_{post},$$ (17)

where $T_{pre}$, $T_{inf}$, and $T_{post}$ denote the preprocessing time, network inference time, and post-processing time, respectively. The throughput is computed from the total latency, as shown in Eq (18),


$$FPS=\frac{1000}{T_{total}},$$ (18)

with $T_{total}$ measured in milliseconds per image.

### Ablation experiments

To evaluate the individual contributions and the collaborative effectiveness of each core component within DPCNet, we conducted a series of incremental ablation experiments on the HIT-UAV dataset. All experiments adopted the same CIoU loss function as used in the YOLO11n baseline to ensure consistency in regression optimization. The YOLO11n model served as the baseline, upon which the DPD head, DPCP block, and DSFI block were progressively integrated. The quantitative results are summarized in Table 1.

**Table 1:** Ablation experiment results on HIT-UAV dataset.

Model	DPD	DPCP	DSFI	Precision	Recall	mAP@0.5	mAP@0.5:0.95	GFLOPs	Parameters/M
YOLO11n				0.785	0.735	0.769	0.493	6.3	2.58
	✓			0.823	0.722	0.787	0.516	6.7	2.85
	✓	✓		0.860	0.722	0.794	0.518	6.9	2.89
	✓	✓	✓	0.820	0.754	0.802	0.526	7.6	1.41

The results indicate that all proposed components contribute significantly to the overall detection performance. When only the DPD head was integrated, the model precision increased from 0.785 to 0.823, and mAP@0.5 improved from 0.769 to 0.787, suggesting that the dual-path design provides task-specialized representations, while the explicit cross-branch interaction further promotes semantic–geometric alignment between classification and regression. With the addition of the DPCP block, the precision further increased to 0.860, and mAP@0.5 rose to 0.794, confirming the effectiveness of the dual-path cross perception mechanism in multi-scale feature fusion and fine detail preservation. When all three components were enabled simultaneously, the model maintained high precision while the recall improved to 0.754, and mAP@0.5 and mAP@0.5:0.95 reached 0.802 and 0.526, respectively. This demonstrates that the DSFI block plays a crucial role in cross-level semantic alignment and complementary feature interaction. In addition, the complete model reduced the number of parameters by approximately 45% compared with the baseline, highlighting its strong lightweight characteristics. Overall, these results demonstrate that DPCNet achieves a well-balanced trade-off between accuracy and efficiency, with all modules complementing and reinforcing one another at different feature levels, thereby providing a robust structural foundation for further performance enhancement.

We conduct a targeted ablation study to examine how cross-branch interaction affects the DPD head. The dual-path decoupled head is kept unchanged, and only the direction of cross-branch information transfer between the classification and regression branches is varied. Three variants are considered: (i) unidirectional transfer from classification to regression (cls→reg), (ii) unidirectional transfer from regression to classification (reg→cls), and (iii) without interaction. Table 2 reports the results of these variants.

**Table 2:** Targeted ablation results of cross-branch interaction in the DPD head on HIT-UAV dataset.

Setting	Precision	Recall	mAP@0.5	mAP@0.5:0.95
cls→reg only	0.828	0.732	0.781	0.507
reg→cls only	0.859	0.702	0.779	0.497
without interaction	0.812	0.722	0.776	0.503

These results indicate that removing cross-branch interaction decreases mAP@0.5:0.95 from 0.516 (the full DPD head) to 0.503. Although the gain is modest, it is consistent with the improvement brought by the complete DPD head reported in Table 1, suggesting that cross-branch interaction benefits localization quality under multiple IoU thresholds. The one-way variants exhibit complementary tendencies. The cls→reg variant increases recall to 0.732 but yields a lower mAP@0.5:0.95 of 0.507, suggesting that semantic cues mainly improve target coverage. In contrast, the regcls variant raises precision to 0.859 but reduces recall to 0.702 and mAP@0.5:0.95 to 0.497, indicating that geometric cues mainly suppress false positives but can overly constrain classification without reciprocal semantic support. Overall, bidirectional interaction combines these two effects and results in the most robust detection performance.

To further investigate the impact of bounding box regression losses on detection performance, additional experiments were conducted within the DPCNet framework by integrating three IoU-based loss functions: DIoU, GIoU, and Shape-IoU. As shown in Table 3, different IoU variants affect precision, recall, and localization accuracy.

**Table 3:** Comparison of bounding box regression loss functions within DPCNet.

Model	DIoU	GIoU	Shape-IoU	Precision	Recall	mAP@0.5	mAP@0.5:0.95
DPCNet	✓			0.861	0.727	0.810	0.528
		✓		0.863	0.743	0.805	0.522
			✓	0.854	0.782	0.820	0.530

DIoU yields precision 0.861, mAP@0.5 0.810, and mAP@0.5:0.95 0.528, indicating a good balance between overlap and center distance. GIoU raises precision to 0.863 but lowers mAP@0.5 to 0.805 and mAP@0.5:0.95 to 0.522; the stronger penalty on regions without overlap increases localization sensitivity for high confidence targets while reducing robustness on small or sparse objects. Shape-IoU achieves the best recall at 0.782 and the highest mAP@0.5 and mAP@0.5:0.95 at 0.820 and 0.530 by enforcing a shape similarity constraint that handles aspect ratio and scale variation. Overall, Shape-IoU is the most stable and adaptable in geometry-aware settings. Given the goal of multi-scale small object detection in complex UAV scenes, Shape-IoU is adopted as the default bounding box regression loss in DPCNet.

### Comparison with other methods

To evaluate the performance of DPCNet in infrared UAV scenarios, comparative experiments were conducted on the HIT-UAV test set. The proposed model was benchmarked against several widely recognized lightweight detection frameworks, including YOLOv5n, YOLOv8n, YOLOv10n [54], YOLO11n, and FBRT-YOLO [55]. All models were trained and evaluated under the same experimental environment and configuration, using identical dataset splits. The YOLO-n variants (e.g., v5n/v8n/v10n/11n) are representative nano-scale configurations across different generations of the YOLO family and are widely used as strong real-time baselines for edge deployment, making them suitable references for lightweight UAV perception. Moreover, FBRT-YOLO is a newly proposed aerial-image detector that targets UAV scenarios, which improves multi-scale feature representation and small-object perception through lightweight modules while maintaining high efficiency. The results of these comparisons are summarized in Table 4.

**Table 4:** Comparison of detection performance on the HIT-UAV test set.

Model	Precision	Recall	mAP@0.5	mAP@0.5:0.95
YOLOv5n	0.758	0.703	0.746	0.465
YOLOv8n	0.805	0.741	0.786	0.497
YOLOv10n	0.839	0.702	0.781	0.509
YOLO11n	0.785	0.735	0.769	0.493
FBRT-YOLO	0.841	0.733	0.782	0.515
DPCNet	0.854	0.782	0.820	0.530

DPCNet exhibits notable improvements over all comparison models in both mAP@0.5 and mAP@0.5:0.95. DPCNet achieves an mAP@0.5 of 0.820, representing a 5.1% increase compared with YOLO11n at 0.769. Relative to YOLOv10n, DPCNet shows a 3.9% improvement from 0.781 to 0.820, while surpassing YOLOv8n by 3.4% and YOLOv5n by 7.4%. Compared with FBRT-YOLO, DPCNet attains a 3.8% gain, rising from 0.782 to 0.820. For mAP@0.5:0.95, DPCNet reaches 0.530, which is 3.7% higher than YOLO11n‘s 0.493 and 6.5% higher than YOLOv5n‘s 0.465. It also improves upon YOLOv8n by 3.3%, YOLOv10n by 2.1%, and FBRT-YOLO by 1.5%. In addition, DPCNet surpasses all other models in both precision and recall, highlighting its strength in small-object detection across diverse UAV imaging conditions.

Further evaluation was conducted on the VisDrone2019 dataset to assess DPCNet‘s generalization capability under visible-light UAV scenes. As shown in Table 5, DPCNet achieves a precision of 0.408 and a recall of 0.303, representing improvements of 2.9% and 1.3%, respectively, compared with YOLO11n. The mAP@0.5 increases by 2.0%, reaching 0.285, while mAP@0.5:0.95 improves by 1.4%, reaching 0.162. Compared with other models, DPCNet maintains overall superior performance, confirming its effectiveness in detecting small-object targets within complex and cluttered visible-light UAV imagery.Overall, the performance improvement of DPCNet on the VisDrone2019 dataset aligns closely with its results on the infrared dataset, indicating that the model achieves stable detection performance across different imaging modalities and environmental conditions. This consistency verifies that DPCNet provides reliable and efficient perception support for multi-scenario UAV vision applications.

**Table 5:** Comparison of detection performance on the VisDrone2019 test set.

Model	Precision	Recall	mAP@0.5	mAP@0.5:0.95
YOLOv5n	0.371	0.287	0.258	0.144
YOLOv8n	0.388	0.291	0.266	0.149
YOLOv10n	0.391	0.286	0.266	0.148
YOLO11n	0.379	0.290	0.265	0.148
FBRT-YOLO	0.384	0.298	0.272	0.155
DPCNet	0.408	0.303	0.285	0.162

Inference efficiency is critical for practical UAV deployment, as it directly affects real-time responsiveness and the feasibility of long-duration or large-area operations. To characterize efficiency in both research and deployment settings, we report runtime under two widely used inference configurations: (i) *PyTorch GPU inference*, which serves as the default framework for model development and reproducible evaluation, and (ii) *TensorRT FP16 inference*, which represents a deployment-oriented optimized engine leveraging reduced-precision execution and runtime optimizations. Importantly, the two measurements are obtained under different evaluation scopes. The PyTorch runtime corresponds to the model forward pass, whereas the TensorRT results are measured using an end-to-end pipeline that includes preprocessing, network inference, and post-processing. Accordingly, they are reported as complementary indicators of efficiency rather than strictly comparable metrics. This distinction is particularly relevant because, for very lightweight models, the E2E latency can be largely influenced by preprocessing/post-processing and runtime overhead, while models with higher computational complexity typically benefit more from optimized inference backends.

All tests are conducted at a fixed input resolution of 640×640 with single-image inference. Table 6 summarizes the runtime results, including the E2E breakdown for TensorRT FP16.

**Table 6:** Runtime comparison between DPCNet and YOLO11n.

Model	PyTorch GPU (ms, FPS)	E2E Pre (ms)	E2E Inf (ms)	E2E Post (ms)	E2E Total (ms/image, FPS)
DPCNet	32.45, 30.81	8.13	3.94	2.19	14.26, 70.15
YOLO11n	5.91, 169.08	8.24	0.94	1.49	10.67, 93.69

Under PyTorch GPU inference, DPCNet requires 32.45 ms/image (30.81 FPS), while YOLO11n achieves 5.91 ms/image (169.08 FPS). Under TensorRT FP16 with the E2E pipeline, DPCNet attains 14.26 ms/image (70.15 FPS), consisting of 8.13 ms preprocessing, 3.94 ms inference, and 2.19 ms post-processing. YOLO11n reaches 10.67 ms/image (93.69 FPS), with 8.24 ms preprocessing, 0.94 ms inference, and 1.49 ms post-processing. Overall, the E2E breakdown indicates that preprocessing and post-processing contribute a substantial portion of the total latency for both models, whereas the optimized inference backend mainly reduces the network inference component.

However, end-to-end inference latency remains an important consideration for practical deployment, and DPCNet is more sensitive due to its higher computational complexity. This motivates future deployment-oriented optimizations at both the model and system levels, including architectural simplification and operator fusion, as well as leveraging optimized inference engines and deployment backends when available. In addition, streamlining the preprocessing and post-processing pipeline and applying model compression techniques such as pruning and quantization can further reduce runtime overhead. Future work will explore these directions to jointly improve efficiency and accuracy, thereby better supporting real-time UAV applications in complex environments.

### Visual analysis

To further validate the detection effectiveness of DPCNet under real-world, complex UAV conditions, a visual comparison was conducted on representative image samples from the HIT-UAV and VisDrone2019 datasets. The visualization results were compared with those of the baseline model YOLO11n, focusing on small object detection, localization accuracy, background suppression, and separation of densely distributed targets.

**Fig 5:**
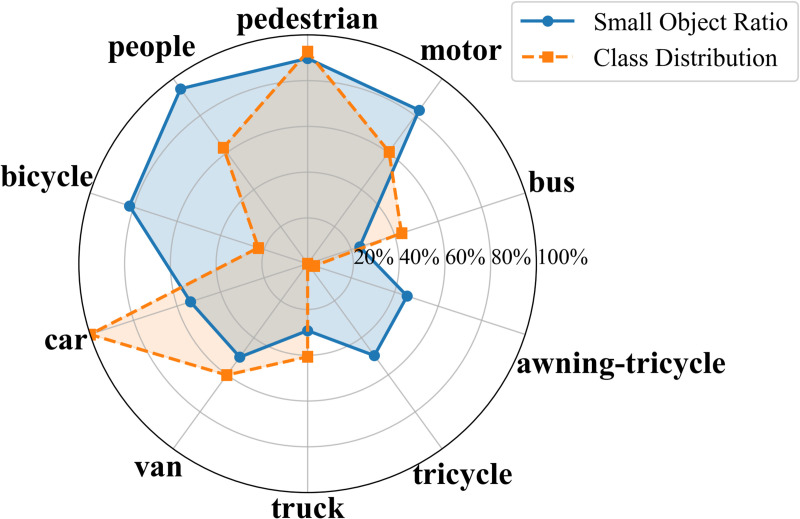
Radar chart of small-object proportions in the VisDrone2019 dataset.

**Fig 6:**
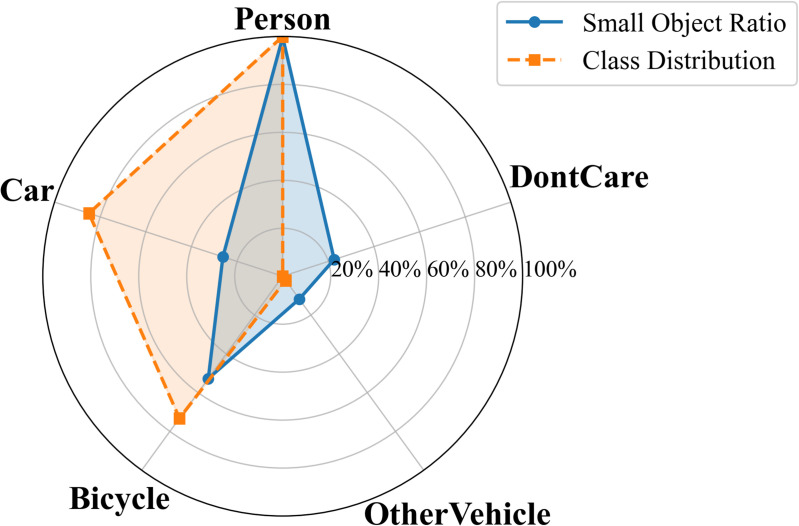
Radar chart of small-object proportions in the HIT-UAV dataset.

In UAV aerial imagery, the presence of small objects is one of the main factors limiting detection performance. According to the standard definition, an object is considered small when its area is less than 32×32 pixels. To further illustrate the structural distribution of small objects across categories, radar charts were generated for both datasets, as shown in Fig 5 and Fig 6. The category proportions were normalized, and the ratios of small objects were plotted on a logarithmic scale to emphasize magnitude differences among categories.

The results show that small objects not only dominate the total number of instances but also display a highly concentrated distribution across multiple classes. In the VisDrone2019 dataset, small objects account for 89.73% and 94.49% in the pedestrian and people categories, respectively. The motor and bicycle classes contain 82.93% and 81.95%, while the car and van categories include 53.86% and 50.46%. Even for larger categories such as truck and bus, small objects still constitute approximately 20–30% of total samples. In the HIT-UAV dataset, this concentration is even more pronounced: the person category contains 99.69% small objects, the bicycle category accounts for 52.76%, and although cars are generally larger targets, small instances still represent 26.21%.

**Fig 7:**
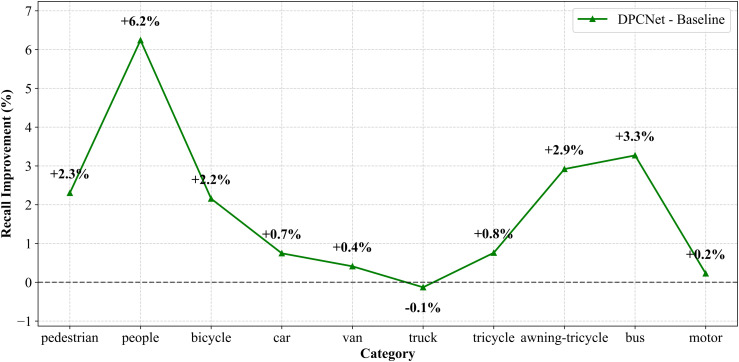
Recall-rate difference curves for small objects across categories on VisDrone2019.

**Fig 8:**
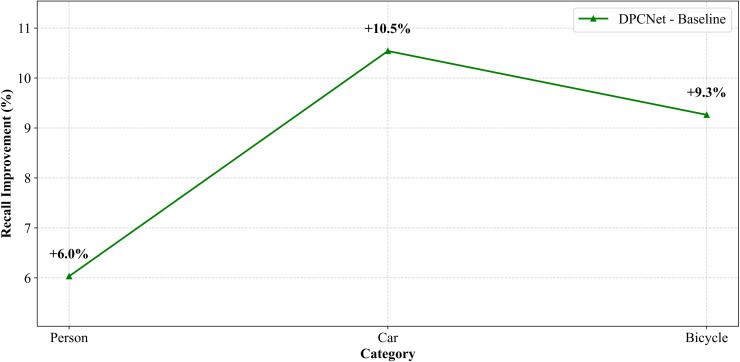
Recall-rate difference curves for small objects across categories on HIT-UAV.

To evaluate DPCNet‘s performance in small-object detection, experiments were conducted on two UAV vision datasets: VisDrone2019 and HIT-UAV. Both datasets define small objects as targets smaller than 32×32 pixels, with an IoU threshold of 0.2. The confidence thresholds were set to 0.001 for VisDrone2019 and 0.75 for HIT-UAV according to dataset noise characteristics, as shown in Fig 7 and Fig 8.

To reduce statistical bias, analysis focused on categories containing a substantial proportion of small objects, such as pedestrians, people, and bicycles, while excluding categories with low ratios of small targets. On the VisDrone2019 dataset, recall for small objects increased from 47.6% to 49.6% with DPCNet. Notable improvements were observed for densely distributed targets: pedestrian recall rose from 42.6% to 44.9%, people from 14.1% to 20.3%, and tricycles from 12% to 14.2%. The awning tricycle category exhibited the highest improvement, with recall increasing by 43.9%, from 6.6% to 9.5%. These results demonstrate that DPCNet enhances small-object detection in complex, cluttered UAV environments. On the HIT-UAV dataset, recall improved from 20.9% to 27.8% under low illumination conditions. Specifically, recall for persons increased from 20.2% to 26.2%, for cars from 36.5% to 47.0%, and for bicycles from 13% to 22.3%. These results confirm that DPCNet effectively preserves fine-scale features and suppresses background noise, even in thermal infrared imagery.

**Fig 9:**
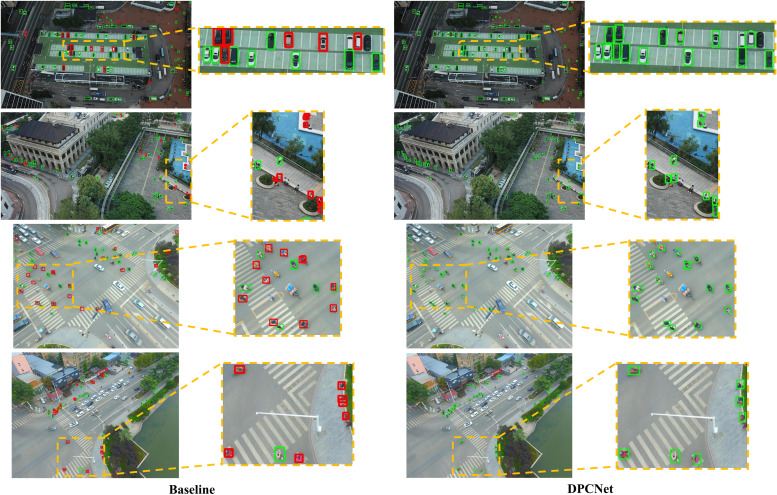
Small-object detection comparison on VisDrone2019.

**Fig 10:**
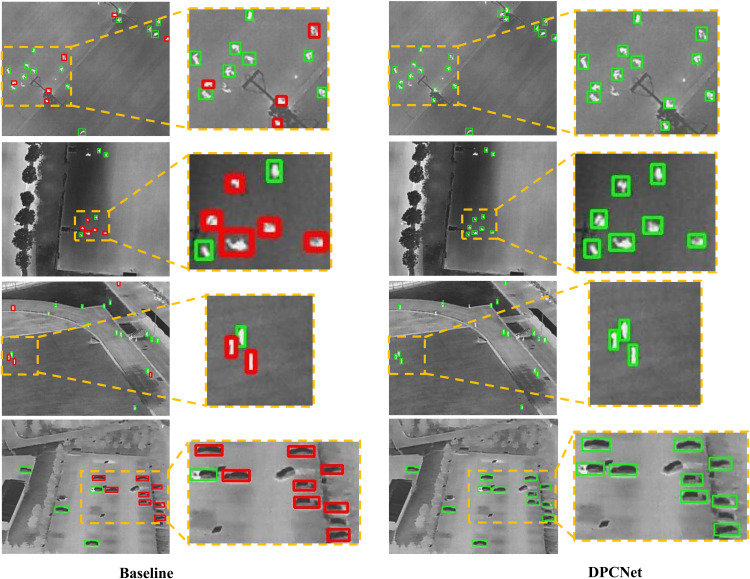
Small-object detection comparison on HIT-UAV.

Fig 9 and Fig 10 provide a visual comparison of small-object detection between the baseline model and DPCNet. In the baseline results, small objects are frequently missed or inaccurately localized, whereas DPCNet successfully detects a wider range of fine-grained targets with improved spatial precision. These visual observations are consistent with the quantitative analysis, confirming that DPCNet‘s enhanced feature interaction and multi-scale perception mechanisms substantially improve small-object detection in complex UAV imagery.

**Fig 11:**
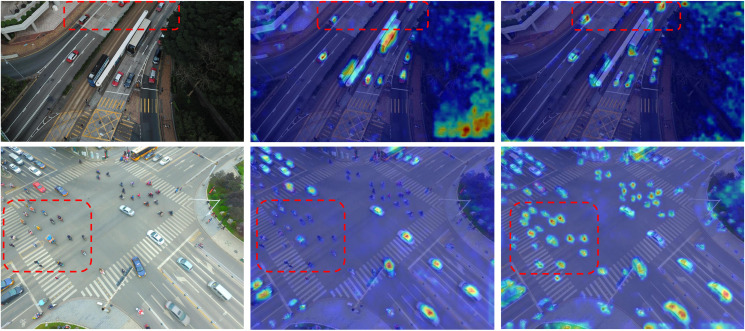
Heatmaps for small-object detection on VisDrone2019.

**Fig 12:**
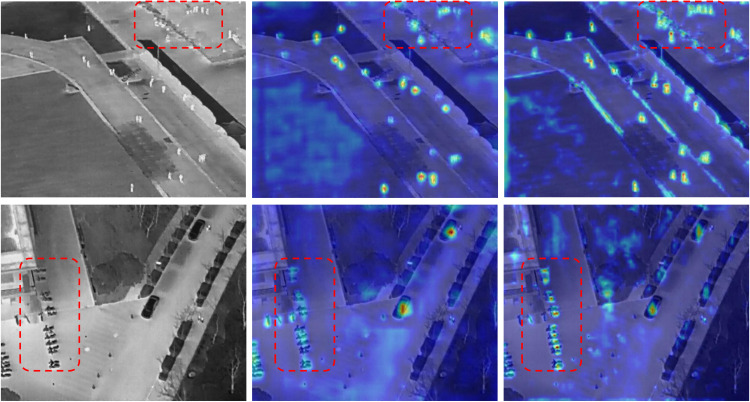
Heatmaps for small-object detection on HIT-UAV.

We further present heatmaps focusing on small-object targets in Fig 11 and Fig 12. For each dataset, two scenes are randomly selected and ordered from left to right as the original image, the baseline heatmap, and the DPCNet heatmap. Red dashed boxes highlight regions with clear improvements. Compared with the baseline, DPCNet produces more concentrated and sharper responses on small targets while effectively suppressing background activation, especially in crowded or low-texture regions. The consistent patterns observed across VisDrone2019 and HIT-UAV confirm that DPCNet allocates attention more precisely to small objects.

Further analysis indicates that the details revealed in the heatmaps align well with the design rationale of the proposed modules. The Dual Path Cross Perception block with the Spatial Guided Gate (SGG) and Feature Statistics Channel Gate (FSCG) calibrates spatial and channel attention toward task-relevant regions while attenuating background clutter, resulting in cleaner activation maps and reduced responses to foliage, road markings, and thermal shadows. The Deep and Shallow Feature Interaction block aligns hierarchical representations and constructs a similarity-based mask with an adaptive threshold, concentrating activation near object centers, producing coherent short-range traces along lanes and pedestrian queues, and reweighting high-resolution feature maps. The Dual Path Decoupled Detection Head introduces cross-channel and spatial guidance that strengthens the correspondence between classification cues and localization geometry, yielding sharper activation peaks on small targets and reducing spillover into coarse features across both datasets.

**Fig 13:**
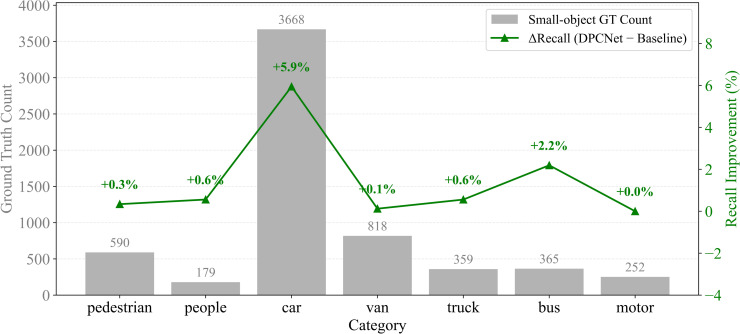
Recall improvement for small objects under low illumination on VisDrone2019.

To systematically evaluate the robustness of DPCNet under low-illumination conditions, a nighttime subset was constructed from the VisDrone2019 dataset. This subset contains 326 images, accounting for 20% of the test set, with a brightness threshold below 30. Quantitative analyses were conducted on small objects, defined as those with areas smaller than 32×32 pixels, and the recall rate differences across categories were plotted, as shown in Fig 13, where gray bars represent the number of objects. The tricycle category, which appeared only once in the nighttime subset, was excluded from the small object analysis.

In the nighttime small object subset, DPCNet achieved an overall recall of 28.1%, an absolute improvement of 3.8% compared with the baseline recall of 24.3%. The improvement was most significant in categories with high or moderate object density. For example, recall for cars, which account for 58.9% of all small-object instances, increased from 35.7% to 41.6%, an absolute gain of 5.9%. Similarly, recall for buses improved from 6.6% to 8.8%, and for trucks from 3.1% to 3.7%. The recall gains for pedestrians and motor vehicles were relatively modest, with pedestrians increasing by 0.3% and motor vehicles maintaining comparable performance to the baseline. Notably, in the sparse and challenging *people* category, DPCNet successfully detected small objects for the first time, achieving a recall of 0.6%, demonstrating its capability to handle difficult cases.

**Fig 14:**
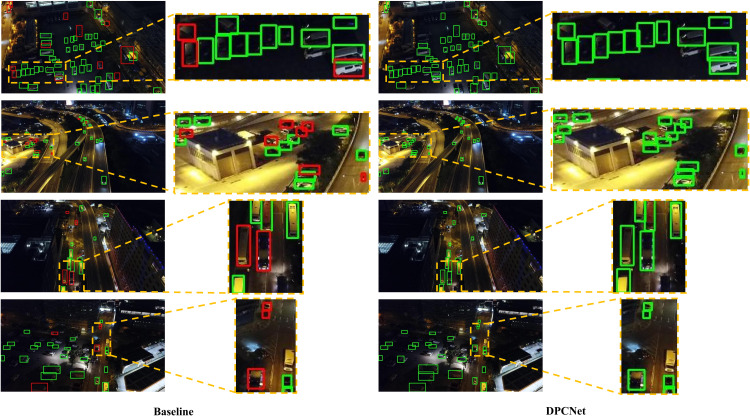
Visual comparison of small-object detection under low illumination.

Fig 14 visually compares detection results, with the left panel showing the baseline model‘s predictions and the right panel displaying DPCNet‘s enhanced detection outcomes. The results confirm that DPCNet, equipped with specialized feature enhancement mechanisms, effectively addresses key challenges associated with nighttime UAV imaging, including low illumination, weak contrast, and noise interference. The model exhibits strong robustness in detecting small objects under complex low-illumination conditions and shows particular superiority in vehicle-related categories, which are essential for UAV-based traffic perception and situational awareness.

To comprehensively evaluate the robustness of DPCNet in real UAV vision tasks, three challenging subsets were constructed from the VisDrone2019 test set, targeting high object density, scale variation, and object overlap. The dense scene subset contains images with more than 50 small objects whose areas are smaller than 32×32 pixels. The scale variation subset includes images where the ratio between the equivalent side lengths of the largest and smallest objects exceeds 10; the equivalent side length is defined as $\sqrt{wh}$, where *w* and *h* denote the width and height of the object. The overlap subset consists of images that include at least one pair of small objects with Intersection over Union greater than 0.5. These subsets contain 269, 733, and 202 images, respectively, and represent typical challenges in large-scale aerial surveillance.

**Fig 15:**
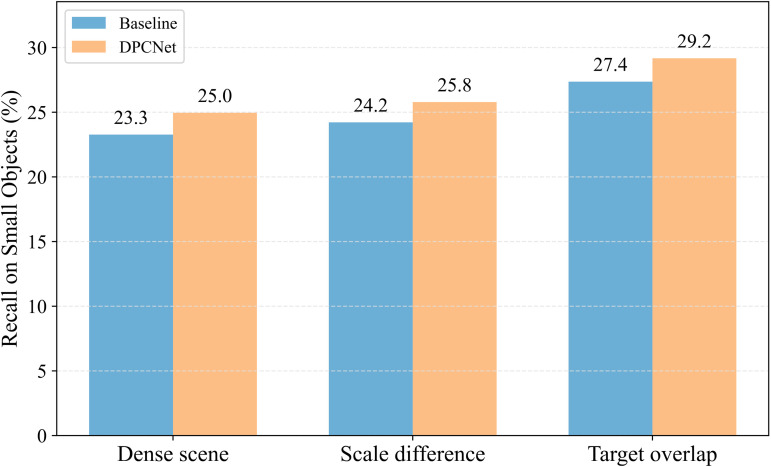
Recall comparison in three challenging subsets.

Fig 15 compares the small object recall of YOLO11n and DPCNet. In dense scenes with 26,497 instances, DPCNet achieves a recall of 25.0% compared with 23.3% for the baseline, a gain of 1.7%. In the scale variation subset with 34,436 instances, DPCNet attains 25.8% compared with 24.2%, an improvement of 1.6%. In the overlap subset with 14,106 instances, DPCNet reaches 29.2% compared with 27.4%, an increase of 1.8%. These results indicate that DPCNet provides stronger feature discrimination in crowded regions, better cross-scale representation, and more accurate localization under occlusion and boundary ambiguity.

**Fig 16:**
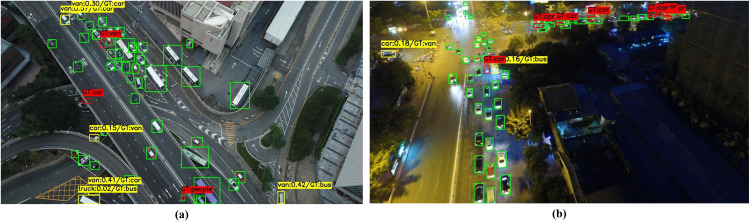
Representative failure cases of DPCNet. **(a)** Dense daytime traffic scene. **(b)** Low-illumination night scene.

To further examine the limitations of DPCNet beyond the overall quantitative gains, representative failure cases were summarized to provide useful insights for future research. Fig 16 presents two typical scenarios, where green boxes denote correct predictions, yellow boxes indicate classification errors, and red boxes represent missed targets. Fig 16(a) shows a dense daytime traffic scene in which numerous small vehicles appear with severe occlusion and spatial overlap under top-down viewpoints, significantly compressing fine-grained appearance cues and object boundaries. As a result, DPCNet may still confuse visually similar categories such as *car* and *van*, occasionally misclassify large vehicles (e.g., *bus*/ *truck*) into neighboring vehicle classes, and miss a small portion of tiny instances in highly crowded regions. Fig 16(b) illustrates a low-illumination night scene with glare and noise, where weakened object–background contrast suppresses discriminative responses, leading to missed detections of small and distant targets as well as sporadic low-confidence misclassifications. These observations suggest that future improvements could focus on enhancing fine-grained vehicle discrimination, integrating contrast/illumination-aware feature enhancement, and adopting stronger instance separation strategies for densely distributed small-object targets.

Overall, DPCNet improves recall by approximately 1.6%–1.8% across all three subsets, confirming robust performance in scenarios characterized by high density, large scale variation, and severe occlusion, while extremely crowded layouts and low contrast conditions remain challenging, and supporting its application in real-world UAV visual tasks.

## Conclusion

This study proposes DPCNet, a single-stage, end-to-end detector for small-object detection in UAV imagery. The network integrates the Dual Path Cross Perception (DPCP) block, the Deep and Shallow Feature Interaction (DSFI) block, the Dual Path Decoupled Detection Head (DPD Head), and a geometry-sensitive Shape-IoU loss to improve recognition and localization of tiny, dense, and occluded targets while maintaining a compact architecture. On the VisDrone2019 and HIT-UAV datasets, DPCNet achieves consistent gains over YOLO11n, with mAP@0.5 increasing by 2.0% and 5.1%, respectively, alongside higher precision and recall. Subset analyses further indicate improved robustness in challenging settings, including nighttime scenes, crowded layouts, large-scale variation, and object overlap. With only moderate computational overhead, the parameter count is reduced by approximately 45%, confirming the efficiency of the proposed design. Moreover, DPCNet maintains real-time end-to-end throughput on GPU under TensorRT FP16, supporting practical deployment. Future work will extend validation to a broader range of UAV scenarios and adverse conditions, accelerate inference for edge deployment, and explore multimodal inputs and temporal information to further enhance robustness and generalization.
